# Lower limb biomechanics during running in individuals with achilles tendinopathy: a systematic review

**DOI:** 10.1186/1757-1146-4-15

**Published:** 2011-05-30

**Authors:** Shannon E Munteanu, Christian J Barton

**Affiliations:** 1 Musculoskeletal Research Centre, Faculty of Health Sciences, La Trobe University, Bundoora 3086, Victoria, Australia; 2 Department of Podiatry, Faculty of Health Sciences, La Trobe University, Bundoora 3086, Victoria, Australia; 3 School of Physiotherapy, Faculty of Health Sciences, La Trobe University, Bundoora 3086, Victoria, Australia

**Keywords:** Achilles tendon, Tendinopathy, Biomechanics, Risk factor

## Abstract

**Background:**

Abnormal lower limb biomechanics is speculated to be a risk factor for Achilles tendinopathy. This study systematically reviewed the existing literature to identify, critique and summarise lower limb biomechanical factors associated with Achilles tendinopathy.

**Methods:**

We searched electronic bibliographic databases (Medline, EMBASE, Current contents, CINAHL and SPORTDiscus) in November 2010. All prospective cohort and case-control studies that evaluated biomechanical factors (temporospatial parameters, lower limb kinematics, dynamic plantar pressures, kinetics [ground reaction forces and joint moments] and muscle activity) associated with mid-portion Achilles tendinopathy were included. Quality of included studies was evaluated using the Quality Index. The magnitude of differences (effect sizes) between cases and controls was calculated using Cohen's d (with 95% CIs).

**Results:**

Nine studies were identified; two were prospective and the remaining seven case-control study designs. The quality of 9 identified studies was varied, with Quality Index scores ranging from 4 to 15 out of 17. All studies analysed running biomechanics. Cases displayed increased eversion range of motion of the rearfoot (d = 0.92 and 0.67 in two studies), reduced maximum lower leg abduction (d = -1.16), reduced ankle joint dorsiflexion velocity (d = -0.62) and reduced knee flexion during gait (d = -0.90). Cases also demonstrated a number of differences in dynamic plantar pressures (primarily the distribution of the centre of force), ground reaction forces (large effects for timing variables) and also showed reduced peak tibial external rotation moment (d = -1.29). Cases also displayed differences in the timing and amplitude of a number of lower limb muscles but many differences were equivocal.

**Conclusions:**

There are differences in lower limb biomechanics between those with and without Achilles tendinopathy that may have implications for the prevention and management of the condition. However, the findings need to be interpreted with caution due to the limited quality of a number of the included studies. Future well-designed prospective studies are required to confirm these findings.

## Background

Achilles tendinopathy is a common musculoskeletal disorder that can impair physical function in daily living, occupation and sporting environments. The prevalence of Achilles tendinopathy has been reported to be greater in males [1]. The condition accounts for between 8 and 15% of all injuries in recreational runners [2-4] and has a cumulative lifetime incidence of approximately 24% in athletes [5]. Although Achilles tendinopathy is common in athletes, one-third of patients with chronic Achilles tendinopathy are not physically active [6]. In some settings, approximately 30% of patients who present with this condition undergo surgical treatment [6,7].

Achilles tendinopathy is considered a multifactorial condition, with both extrinsic and intrinsic factors thought to contribute to its development [8-10]. Proposed extrinsic risk factors include altered weightbearing surfaces (excessively hard, slippery or uneven) [8,10], inappropriate footwear [8,10,11], training errors [10], use of specific medications such as fluoroquinolones [12] and the type of exercise activity (e.g., sports involving the stretch-shorten cycle such as running or jumping) [5]. Proposed intrinsic risk factors include previous injury [8], increased age [13], presence of specific genetic variations such as polymorphisms occurring within the COL5A1 and tenascin-C genes [14], male gender [15], increased adiposity and/or metabolic disorders [16,17], pre-existing tendon abnormalities [18], triceps surae inflexibility [10,19], hormonal status [20-22] and abnormal lower limb biomechanics [8,10,15,23].

Alterations in lower limb biomechanical characteristics including temporospatial parameters, lower limb kinematics, dynamic plantar pressures, kinetics (ground reaction forces and joint moments) and muscle activity are frequently associated with Achilles tendinopathy [8,15,23]. One biomechanical factor commonly considered to be associated with Achilles tendinopathy is the presence of excessive foot pronation [8]. Clement et al. [10] originally proposed that excessive pronation of the foot may lead to Achilles tendinopathy through two mechanisms. First, excessive pronation of the foot is speculated to create greater hindfoot eversion motion, resulting in excessive forces on the medial aspect of the tendon and subsequent microtears. Second, abnormal pronation of the foot is thought to lead to asynchronous movement between the foot and ankle during the stance phase of gait, resulting in a subsequent 'wringing' effect within the Achilles tendon. This 'wringing' effect is theorised to cause vascular impairment within the tendon and peritendon [10] and elevated tensile stress [24] leading to subsequent degenerative changes in the Achilles tendon. In addition to kinematic theories, altered lower limb muscle function (timing, amplitude or co-ordination of contractions of the triceps surae) [23-26] and altered lower limb kinetics [11,24,25,27] have also been speculated to be risk factors for Achilles tendinopathy by increasing tendon loading.

Several studies have been performed to investigate the association between abnormal lower limb biomechanics and Achilles tendinopathy. Critiquing and summarising results from these studies is now required to assist in the development of; (i) preventative strategies, and; (ii) specific and effective management strategies for the condition. However, at present, the aetiology of Achilles tendinopathy is not clearly understood [8]. Therefore, the aim of the present study was to perform a systematic review of the existing literature (prospective cohort and retrospective case-control studies) to identify, critique and summarise lower limb biomechanical factors associated with Achilles tendinopathy.

## Methods

### Inclusion and exclusion criteria

Prospective cohort and case-control studies evaluating biomechanical factors associated with mid-portion Achilles tendinopathy (i.e., 2-6 cm proximal to its insertion) were considered for inclusion. The inclusion criteria required participants to be described as having: *midsubstance tendinopathy of the Achilles, Achilles tendinitis, tenosynovitis or tendinosis *[28]. Additional terms such as *Achilles tendinopathy, tenopathy, tendinosis, partial rupture, paratenonitis, tendovaginitis, peritendinitis and achillodynia *have also been used to describe the problems of non-insertional pain associated with the Achilles tendon so were also used [29]. Measures of interest were gait characteristics including temporospatial parameters, lower limb kinematics, dynamic plantar pressures, kinetics (ground reaction forces and joint moments) and muscle activity.

Unpublished studies, case-series studies, non-peer-reviewed publications, intervention studies, studies not involving humans, reviews, letters, opinion articles, non-English articles and abstracts were excluded. Studies which included participants with concomitant injury or pain from structures other than the mid-portion of the Achilles tendon (e.g., insertional Achilles tendon pathology) or that failed to localise the pathology in the tendon were excluded.

### Search Strategy

MEDLINE (OVID) (1950-), EMBASE (1988-), CINAHL (1981-), SPORTDiscus and Current Contents (1993 week 27-) electronic databases were searched in November 2010 (week 3). A generic search strategy was formulated [28,30] and the results are reported in Additional Data File 1.

### Review process

All titles and abstracts found were downloaded into Endnote version XI (Thomson Reuters, Philadelphia, PA) giving a set of 2701 citations. The set was cross-referenced and any duplicates were deleted, leaving a total of 1575 citations. Each title and abstract was evaluated for potential inclusion by two independent reviewers (SEM and CJB) using a checklist developed from the inclusion/exclusion criteria outlined above (see Additional File 2). If insufficient information was contained in the title and abstract to make a decision on a study, it was retained until the full text could be obtained for evaluation. Any disagreements regarding studies were resolved by a consensus meeting between the two reviewers.

### Methodological quality assessment

The methodological quality of each included study was assessed using 16 items (maximum score of 17) of the 'Quality Index' considered relevant for assessing prospective cohort and case-control study designs (Table 1) [31]. The original Quality Index scale consisting of 26 items was shown to have high internal consistency (KR-20 = 0.89), test-retest (r = 0.88) and inter-rater (r = 0.75) reliability and high criterion validity (r ≥ 0.85) [31]. Two reviewers (SEM and CJB) applied the quality index to each included study independently, and any scoring discrepancies were resolved through a consensus meeting.

**Table 1:** Modified Downs and Black Quality Index results, and inter-rater reliability for each item and total score

	Prospective (P) or retrospective case-control (R) study	(1) Clear aim/hypothesis	(2) Outcome measures clearly described	(3) Participant characteristics clearly described	(5) Confounding variables (age, gender, BMI/height/weight and participant activity levels) described	(6) Main findings clearly described	(7) Measures of random variability provided	(10) Actual probability values reported	(11) Participants asked to participate representative of entire population	(12) Participants prepared to participate representative of entire population	(15) Blinding of outcome assessor	(16) Analyses performed were planned	(18) Appropriate statistics	(20) Valid and reliable outcome measures	(21) Appropriate case-control matching (same population)	(22) Participants recruited over the same period of time	(25) Adjustment made for confounding factors	Total
Azevedo et al. [27]	R	1	1	1	2	1	1	1	U	U	U	1	1	U	U	U	1	11

Baur et al. [11]	R	1	1	0	0	0	0	0	U	U	U	1	1	U	U	U	U	4

Donoghue et al. [34]	R	1	1	0	1	1	1	0	0	0	U	1	1	U	U	U	0	7

Donoghue et al. [33]	R	1	1	0	1	1	1	1	0	0	U	1	1	U	U	U	0	8

Kaufman et al. [19]	P	1	1	1	1	1	1	1	1	U	1	1	1	U	1	1	0	13

McCrory et al. [25]	R	1	1	0	1	1	1	0	U	U	U	1	1	U	U	U	1	8

Ryan et al. [35]	R	1	0	1	1	1	1	1	U	U	U	1	1	U	1	U	1	10

Williams et al. [24]	R	1	1	1	2	1	1	1	U	U	0	1	1	U	U	U	1	11

Van Ginckel et al. [2]	P	1	1	1	2	1	1	1	1	U	1	1	1	U	1	1	1	15

% agreement		100.0	100.0	100.0	77.8	88.9	88.9	88.9	88.9	88.9	77.8	88.9	88.9	100.0	77.8	100.0	88.9	

Reliability		1.00	1.00	1.00	0.63	0.61	0.61	0.77	0.82	0.74	0.57	Uc	Uc	1.00	0.63	1.00	0.80	0.98 (0.905-0.995)

(For items 1-3, 6, 7, 10-12, 15, 16, 18, 20, 21, 22 and 25)-0: No, 1: Yes, U: Unable to determine (which received a score of 0)

(For item 5)-0: No, 1: Partially, 2: Yes

Abbreviations:

Uc; Results not distributed appropriately for this statistic to be calculated.

### Statistical analysis

Inter-rater reliability of each item of the Quality Index was evaluated using unweighted kappa and percentage agreement statistics, and the overall score was evaluated using the intra-class correlation coefficient (ICC_3,1_) with corresponding 95% confidence intervals (CIs).

Means and standard deviations for all continuous data were extracted and effect sizes (Cohen's d) (with 95% CIs) calculated to allow comparison between each study's results. To allow visual comparison, effect sizes were entered into forest plots. Categorical data (e.g. frequency of foot type) was compared between groups using odds ratios (with 95% CIs) transformed to effect sizes (with 95% CIs) as described by Chinn et al. [32] Calculated effect sizes were considered statistically significant if their 95% CI did not cross zero. If inadequate data were available from original studies to complete effect size calculations, attempts were made via email to contact the study's corresponding author for additional data.

Sample sizes (limbs analysed), the presence or absence of symptoms, participant demographics (gender, age, BMI, mass, height, duration of symptoms and sporting experience) and biomechanical analysis details were also extracted to assist in interpretation of findings.

## Results

Following the search, nine studies were deemed appropriate for inclusion [2,11,19,24,25,27,33-35]. This included two prospective cohort [2,19] and seven case-control study designs [11,24,25,27,33-35]. There were no disagreements amongst reviewers. One study [33] did not contain appropriate data to complete effect size calculations, meaning data extraction (effect size calculations) was performed on a total of eight studies [2,11,19,24,25,27,34,35].

### Quality assessment of included studies

All individual items from the Quality Index scale demonstrated high inter-rater reliability (kappas ≥ 0.57) with percentage agreement ≥ 77.8% (Table 1). The total score obtained from the Quality Index scale demonstrated high inter-rater reliability (ICC_3,1 _= 0.98).

### Additional data

Additional data required to complete effect size calculations was provided by Baur et al. [11]. Additionally, Van Ginckel et al. [2] provided revised data for some reported variables which were reported erroneously in their manuscript.

### Methodological data to assist interpretation of results

Table 2 shows the samples sizes and population characteristics. Table 3 shows the biomechanical analysis details of each of the included studies.

**Table 2:** Sample sizes and population characteristics from each included study

Study	Symptomatic (yes/no)	Sample size (limbs)		Gender (n) (Male/Female)		Mean age ± SD (range) (years)		Mass (kg), height (cm), BMI		Experience: years of sporting activity	
		
		AT	C	AT	C	AT	C	AT	C	AT	C
Azevedo et al. [27]	Yes	21	21	16/5	16/5	41.8 ± 9.7 (NR)	38.9 ± 10.1 (NR)	77.6, 177.8, NR	70.2, 174.3, NR	> 3 years*	

Baur et al. [11]	Yes	16	28	NR	NR	36 ± 9 (NR)*		73, 179, NR*		NR 'experienced'*	

Donoghue et al. [33]	No	12	12	11/1	11/1	38.7 ± 8.1 (NR)	44.3 ± 8.4 (NR)	73.3, 175, NR	79.3, 178, NR	NR	NR

Donoghue et al. [34]	No	11	11	10/1	10/1	39.6 ± 7.7 (NR)	45.2 ± 8.1 (NR)	71.9, 174, NR	77.9, 177, NR	NR	NR

Kaufman et al. [19]	No	17	299	17/0	299/0	22.5 ± 2.5 (NR)*		78.0, 177.0, NR*		2-7 times/week fitness preparation, 73% reported having run or jogged on a regular basis for a period of 3 or more months before reporting to training*	

McCrory et al. [25]	Yes	31	58	NR	NR	38.4 ± 1.8 (NR)	34.5 ± 1.2 (NR)	71.4, 174.5, NR	70.0, 174.5, NR	11.9 ± 1.4	9.6 ± 0.8

Ryan et al. [35]	Yes	27	21	NR	NR	40 ± 7 (NR)	40 ± 9 (NR)	78, 181, NR	71, 177, NR	NR	NR

Van Ginckel et al. [2]	No	10	53	2/8	8/45	38.0 ± 11.35 (NR)	40.0 ± 9.00 (NR)	69.8, 167.1, 24.95	70.0, 168.3, 24.69	0	0

Williams et al. [24]	No	8	8	6/2	5/3	36.0 ± 8.2 (NR)	31.8 ± 9.3 (NR)	67.3, 176, NR	65.6, 170, NR	19.1 ± 7.7	11.0 ± 9.1

Abbreviations:

AT, Achilles tendinopathy group; C, control group; NR, not reported; *, Specified total group characteristics only

**Table 3:** Lower limb biomechanical analyses, gait characteristics and footwear conditions of included studies

Study	Biomechanical variable(s)	Gait characteristics	Footwear condition(s)
Azevedo et al. [27]	Muscle activity (integrated EMG: normalised EMG amplitude as a percentage of root mean square amplitude): tibialis anterior, peroneus longus, lateral gastrocnemius, rectus femoris, biceps femoris and gluteus medius; Kinematics (3D using Vicon^® ^System 370 Version 2.5): sagittal plane hip, knee and ankle joints; Kinetics: anterior-posterior and vertical ground reaction force; Temporospatial parameters (speed, stride length, stride time, stride frequency).	Running Uv, Og	C (neutral running shoe)

Baur et al. [11]	Muscle activity (normalised EMG amplitude to mean amplitude of the entire gait cycle and timing of activity): tibialis anterior, peroneals, lateral head of gastrocnemius, medial head of gastrocnemius, soleus; Kinetics: antero-posterior and vertical ground reaction force; Plantar pressures (Novel Pedar^® ^Mobile system): deviation of the centre of pressure.	Running Cv (12 km/hour), Tm	C (gymnastic shoe that simulates barefoot conditions) and C (standardised marketed reference running shoe

Donoghue et al. [33]	Kinematics (3D: functional data analysis using 3D Qualysis system with Peak Motus™ analysis system): frontal plane rearfoot and lower leg, sagittal plane ankle and knee joints.	Running Cv (~2.8 m/s), Tm	U (own running shoes)

Donoghue et al. [34]	Kinematics (3D Qualysis system with Peak Motus™ analysis system): frontal plane rearfoot and lower leg, sagittal plane ankle and knee joints.	Running Cv (~2.5-2.8 ± 0.2-0.4 m/s), Tm	Unable to determine (as type of footwear not specified) and B

Kaufman et al. [19]	Plantar pressures (Tekscan^® ^in-shoe system): dynamic arch index.	Running Uv, Og	C (military footwear) and B

McCrory et al. [25]	Kinematics (2D Motion Analysis high-speed video camera): frontal plane rearfoot. Kinetics: antero-posterior, medio-lateral and vertical ground reaction forces.	Running Uv ('training pace'), T (kinematics), Og (kinetics)	U (own footwear)

Ryan et al. [35]	Kinematics (3D ViconPeak^® ^system with Bodybuilder 3.6^® ^software): frontal and sagittal plane rearfoot and transverse plane tibia.	Running Uv, Og	B

Van Ginckel et al. [2]	Plantar pressures (RsScan Footscan^® ^pressure plate): multiple variables (temporal data, peak force, force-time integrals, contact time, medio-lateral force ratios and position and deviation of the centre of force).	Running Uv, Og	B

Williams et al. [24]	Kinematics and moments (3D Qualisys motion system with Visual 3-D software): transverse plane tibia relative to foot (tibial motion) and tibia relative to femur (knee motion).	Running Cv, Og (3.35 m/s ± 5%).	B

Abbreviations:

EMG, electromyography; 2D, two-dimensional analysis; 3D, three-dimensional analysis; Cv, controlled velocity; Uv, uncontrolled velocity; Og, overground; Tm, treadmill; C, yes and controlled; U, yes but uncontrolled; B, barefoot.

### Differences in lower limb biomechanics between those with and without Achilles tendinopathy

#### Temporospatial gait characteristics

Four [11,24,33,34] studies controlled gait velocity. Of the remaining five studies [2,19,25,27,35], only one [27] reported temporospatial data, with effect size calculations indicating no differences in velocity, stride length, stride time or stride frequency between cases and controls. Additionally, another study [35] reported that no significant differences in gait velocity were evident between groups but did not present supporting data.

#### Lower limb kinematics

**Figure 1:**
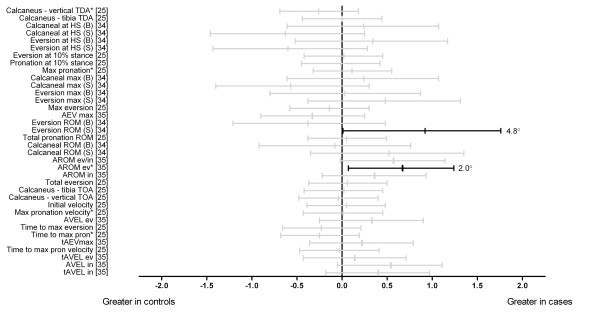
**Frontal plane kinematics of the rearfoot during running (Black plots = significant effects with group difference adjacent the right error bar, Grey plots = non-significant effects)**. *Abbreviations*: Calcaneus-vertical TDA, calcaneus to vertical touch down angle; Calcaneus-tibia TDA, calcaneus to tibia touch down angle; Calcaneal at HS, calcaneal angle (relative to ground) at heel strike; Eversion at HS, angle between rearfoot and lower leg at heel strike; Max pronation, maximum pronation; Calcaneal max, maximum calcaneal angle; Eversion max, maximum eversion; Max eversion, maximum eversion; AEV max, maximum ankle eversion; Eversion ROM, eversion range of motion; Total pronation ROM, total pronation range of motion; Calcaneal ROM, calcaneal angle range of motion; AROM ev/in, total frontal plane range of motion of the ankle; AROM ev, eversion range of motion of the ankle; AROM in, inversion range of motion of the ankle; Calcaneus-tibia TOA, calcaneus to tibia toe-off angle; Calcaneus-vertical TOA, calcaneus-vertical toe-off angle; Max pronation velocity, maximum pronation velocity; AVEL ev, maximum velocity of ankle eversion; Time to max eversion, time to maximum eversion; Time to max pron, time to maximum pronation; tAEVmax, timing of maximum ankle eversion; Time to max pron velocity, time to maximum pronation velocity; tAVEL ev, timing of maximum ankle eversion velocity; AVEL in, maximum velocity of ankle inversion; tAVEL in, timing of maximum ankle inversion velocity; B; barefoot; S, shod. * Variables were reported to have statistically significant differences between groups in original study.

Three studies investigated frontal plane rearfoot kinematics (Figure 1) [25,34,35]. Those with Achilles tendinopathy displayed greater rearfoot eversion range of motion when shod (d = 0.92) but not unshod [34] and greater eversion range of motion of the ankle/rearfoot (d = 0.67) [35]. Effect size calculations for all other frontal plane rearfoot kinematics comparisons were not statistically significant.

**Figure 2:**
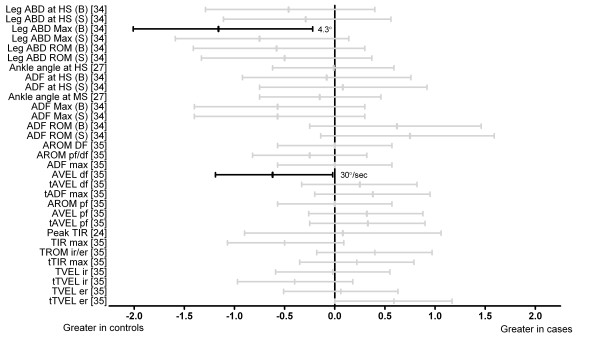
**Kinematics of the tibial segment and ankle during running (Black plots = significant effects with group difference adjacent the right error bar, Grey plots = non-significant effects)**. *Abbreviations*: Leg ABD at HS, leg abduction at heel strike; Leg ABD max, maximum leg abduction; Leg ABD ROM, leg abduction range of motion; Ankle angle at HS, ankle sagittal plane angle at heel strike; ADF at HS, ankle joint dorsiflexion at heel strike; Ankle angle at MS, ankle sagittal plane angle at midstance; ADF Max, maximum ankle joint dorsiflexion; ADF ROM, ankle joint dorsiflexion range of motion; AROM DF, sagittal plane dorsiflexion range of motion of the ankle; AROM pf/df, total sagittal plane motion of the ankle; ADF max, maximum ankle dorsiflexion; AVEL df, maximum dorsiflexion velocity of ankle; tADF max, timing of maximum ankle dorsiflexion; AROM pf, sagittal plane plantarflexion range of motion of the ankle; AVEL pf, maximum plantarflexion velocity of ankle; tAVEL pf, timing of maximum velocity plantarflexion at the ankle; Peak TIR, peak tibial internal rotation; TIR max, maximum tibial internal rotation; TROM ir/er, total transverse tibial range of motion; tTIR max, timing of maximum internal transverse plane tibial rotation; TVEL ir, maximum velocity internal transverse plane tibial rotation; tTVEL ir, timing of maximum velocity internal transverse plane tibial rotation; TVEL er, maximum velocity external transverse plane tibial rotation; tTVEL er, timing of maximum velocity external transverse plane tibial rotation; B, barefoot; S, shod; Sec, seconds.

Four studies investigated tibial segment and ankle joint kinematics (Figure 2) [24,27,34,35]. Donoghue et al. [34] showed reduced maximum lower leg abduction (barefoot) in cases (d = -1.16). Ryan et al. [35] showed reduced maximum ankle dorsiflexion velocity in cases (d = -0.62). All other tibial segment and ankle kinematic comparisons were not significantly different between groups [24,27,34,35].

**Figure 3:**
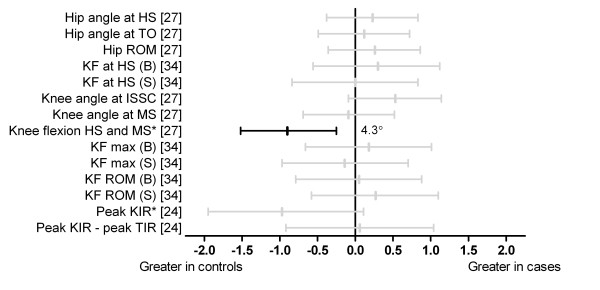
**Kinematics of the hip and knee joints during running (Black plots = significant effects with group difference adjacent the right error bar, Grey plots = non-significant effects)**. *Abbreviations*: Hip angle at HS, sagittal plane hip angle at heel strike; Hip angle at TO, sagittal plane hip angle at toe-off; Hip ROM, sagittal plane hip range of motion; KF at HS, knee flexion at heel strike; Knee angle at ISSC, sagittal plane knee angle at initial supporting surface contact; Knee angle at MS, sagittal plane angle at midstance; Knee flexion HS and MS, knee flexion between heel strike and midstance; KF max, maximum knee flexion; KF ROM, knee flexion range of motion; Peak KIR, peak knee internal rotation; Peak KIR-peak TIR, timing of peak knee internal rotation to peak tibial internal rotation; B, barefoot; S, shod. * Variables were reported to have statistically significant differences between groups in original study.

Three studies performed analyses for knee and hip kinematics (Figure 3) [24,27,34]. Azevedo et al. [27] reported that the magnitude of knee flexion between heel strike and midstance was significantly reduced in cases (d = -0.90). Effect size calculations for all other knee joint kinematics comparisons were not significantly different between groups [24,27,34]. There were no statistically significant effects for comparisons in sagittal plane hip kinematics [27].

#### Plantar pressure parameters

**Figure 4:**
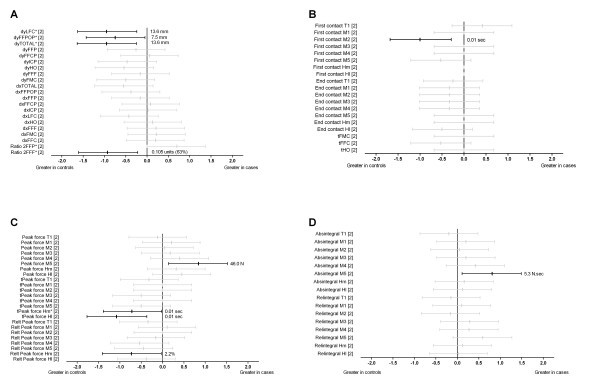
**Dynamic plantar loading variables (plantar pressures) during running (Black plots = significant effects with group difference adjacent the right error bar, Grey plots = non-significant effects)**. Figure 4A shows variables related to the displacement of the centre of force; Figure 4B shows variables related to the timing of loading or unloading of specific regions of the foot; Figure 4C shows variables related to peak force; and Figure 4D shows variables related to the force time integral. *Abbreviations*: dy, displacement of the centre of force in the y-direction (postero-anterior direction); dx, displacement of the centre of force in the x-direction (medio-lateral direction); Ratio2; medio-lateral force distribution underneath the forefoot; First contact, instant at which the zone made contact; End contact, instant at which the zone made end contact; t, time to; Relt; relative time to; Absintegral, absolute force time integral; Relintegral, relative force time integral; LFC, instant of last foot contact; FFPOP, forefoot push-off phase; FFC, instant of first foot contact; FFCP, forefoot contact phase; ICP, initial contact phase; HO: instant of heel off; FFF, instant of forefoot flat; FMC, instant of first metatarsal contact; FFP, foot flat phase; T1, hallux; M1, M2, M3, M4 and M5; metatarsal heads 1 to 5; Hm, medial heel; Hl, lateral heel; N, Newtons; Sec, seconds. * Variables were reported to have statistically significant differences between groups in original study.

**Figure 5:**
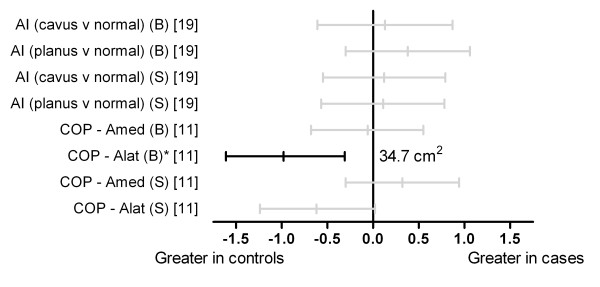
**Differences in the frequency of pes cavus and pes planus and the excursion of the centre of pressure assessed using plantar pressures during running (Black plots = significant effects with group difference adjacent the right error bar, Grey plots = non-significant effects)**. *Abbreviations*: AI, arch index; cavus v normal, frequency of pes cavus to normal foot type; planus v normal, frequency of pes planus to normal foot type; COP-Amed, centre of pressure excursion: medial deviation in relation to the plantar angle; COP-Alat, centre of pressure excursion: lateral deviation in relation to the plantar angle; B, barefoot; S, shod. * Variables were reported to have statistically significant differences between groups in original study.

A large number of plantar pressure parameters were analysed across three studies [2,11,19] (Figures 4A-D and 5). A prospective study by Van Ginckel et al. [2] showed that those who developed Achilles tendinopathy demonstrated significantly reduced displacement of the posterior-anterior component of the centre of force at last foot contact (d = -0.95), posterior-anterior displacement of the centre of force during forefoot push-off phase (d = -0.75), total posterior-anterior displacement of the centre of force (d = -0.95) and medio-lateral force distribution under the metatarsal heads at forefoot flat (d = -0.93) (Figure 4A). Further those who developed Achilles tendinopathy displayed reduced timing of initial contact at the second metatarsal head region (d = -1.00) (Figure 4B), relative peak force at the medial heel (d = -0.73), time to peak force at the lateral heel (d = -1.08) and at the medial heel (d = -0.72) regions (Figure 4C). Additionally, increases were found for peak force at the fifth metatarsal head region (d = 0.84) (Figure 4C) and force-time integral at the fifth metatarsal head region (d = 0.81) (Figure 4D) in those who developed Achilles tendinopathy [2].

Figure 5 shows that lateral deviation of the centre of pressure in the rear-and mid-foot (Alat [barefoot]) was significantly reduced in cases (d = -0.98) [11]. The frequency of dynamic pes planus or pes cavus (assessed using dynamic arch index in both barefoot and shod conditions) was not significantly different between those who did and did not develop Achilles tendinopathy [19].

#### Lower limb external kinetics

**Figure 6:**
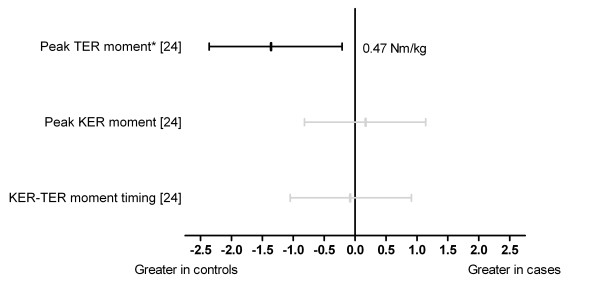
**Lower limb moments during running (Black plots = significant effects with group difference adjacent the right error bar, Grey plots = non-significant effects)**. *Abbreviations*: Peak TER moment, peak tibial external rotation moment; Peak KER moment, peak knee external rotation moment; KER-TER moment timing, knee external rotation moment to tibial external rotation moment timing difference; Nm/kg, Newton metres/kg. * Variables were reported to have statistically significant differences between groups in original study.

One study analysed lower limb joint moments (Figure 6). Peak tibial external rotation moment was significantly reduced in cases (d = -1.29) [24].

**Figure 7:**
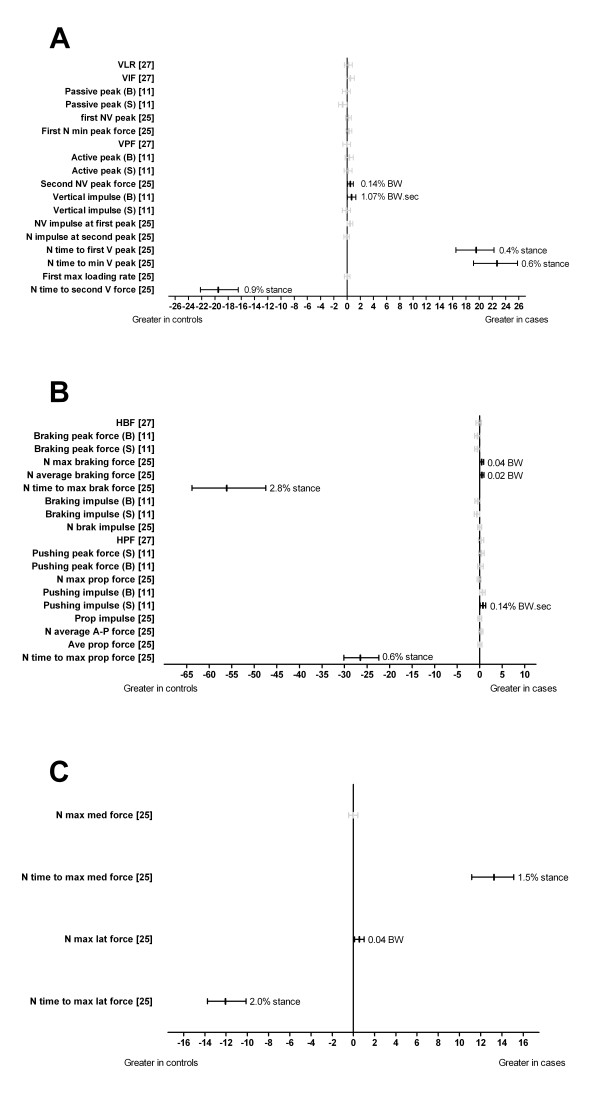
**Ground reaction forces during running (Black plots = significant effects with group difference adjacent the right error bar, Grey plots = non-significant effects)**. Panels A, B and C show vertical, antero-posterior and medio-lateral components respectively. *Abbreviations*: *A*: VLR, vertical loading rate; VIF, vertical impact force peak; Passive peak, passive peak; First NV peak, first normalised vertical peak force; First N min peak force, first normalised minimum peak force; VPF, vertical propulsive force; Active peak, active peak; Second NV peak force, second normalised vertical peak force; NV impulse at first peak, normalised vertical impulse at first peak; N impulse at second peak, normalised vertical impulse at second peak; N time to first V peak, normalised time to first vertical peak force; N time to min V peak, normalised time to minimum vertical peak force; First max loading rate, first maximum loading rate; N time to second V force, normalised time to second vertical peak force; BW, bodyweight; Sec, seconds. *B: *HBF, Horizontal braking force; N max braking force, normalised maximum braking force; N average braking force, normalised average braking force; N time to max brak force, normalised time to maximum braking force; N brak impulse, normalised braking impulse; HPF, horizontal propulsive force; N max prop force, normalised maximum propulsive force; Prop impulse, propulsive impulse; N average A-P force, normalised average antero-posterior force; Ave prop force, average propulsive force; N time to max prop force, normalised time to maximum propulsive force; BW, bodyweight; Sec, seconds. *C: *N max med force, normalised maximum medial force; N time to max med force, normalised time to maximum medial force; N max lat force, normalised maximum lateral force; N time to max lat force, normalised time to maximum lateral force; BW, bodyweight. * Variables were reported to have statistically significant differences between groups in original study.

Three studies analysed ground reaction forces [11,25,27] (Figure 7A-C). The normalised time to first vertical peak (d = 19.54) [25] and normalised time to minimum vertical peak (d = 22.69) [25] were significantly increased (delayed) in cases (Figure 7A). The normalised time to second vertical force (d = -19.50) [25] was significantly reduced (earlier) in cases (Figure 7A). The second normalised vertical peak force (d = 0.52) [25] and the vertical impulse (barefoot) were significantly increased in cases (d = 0.70) (Figure 7A) [11].

The normalised time to maximum braking force (d = -56.1) [25] and normalised time (% stance) to maximum propulsive force (d = -26.5) [25] were significantly reduced (earlier) in cases (Figure 7B). The normalised maximum braking force (d = 0.46) [25], normalised average braking force (d = 0.52) [25] and pushing impulse (shod) (d = 0.74) [11] were significantly increased in cases (Figure 7B).

The normalised time to maximum lateral force was significantly reduced (earlier) (d = -12.05) [25] and normalised time to maximum medial force was significantly increased (delayed) (d = 13.25) [25] in cases (Figure 7C). The normalised maximum lateral force was significantly increased (d = 0.57) [25] in cases (Figure 7C).

#### Lower limb muscle function

**Figure 8:**
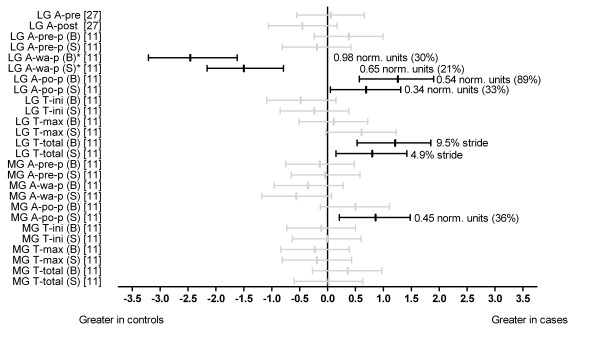
**Function of gastrocnemius during running (Black plots = significant effects with group difference (actual units and as a percentage) adjacent the right error bar, Grey plots = non-significant effects)**. *Abbreviations*: LG, lateral gastrocnemius; MG, medial gastrocnemius; A-pre, amplitude at 100ms pre-heel strike; A-post, amplitude at 100ms post-heel strike, A-pre-p, amplitude during pre-activation phase; A-wa-p, amplitude during weight acceptance phase; A-po-p, amplitude during push-off phase; T-ini, time of start of activation; T-max, time of maximum activation; T-total, total time of activation; B, barefoot; S, shod; Norm. units, normalised to mean amplitude during the entire gait cycle. * Variables were reported to have statistically significant differences between groups in original study.

**Figure 9:**
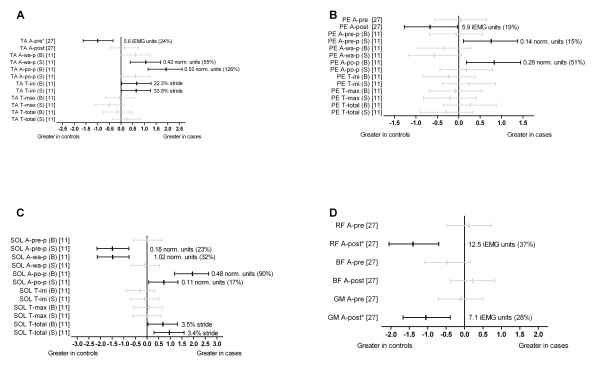
**Function of tibialis anterior (panel A), peroneus longus (panel B), soleus (panel C), as well as rectus femoris, biceps femoris and gluteus medius muscles (panel D) during running (Black plots = significant effects with group difference (actual units and as a percentage) adjacent the right error bar, Grey plots = non-significant effects)**. *Abbreviations*: TA, tibialis anterior; PE, peroneus longus; SOL, soleus; RF, rectus femoris; BF, biceps femoris; GM, gluteus medius; A-pre, amplitude at 100ms pre-heel strike; A-post, amplitude at 100ms post-heel strike, A-pre-p, amplitude during pre-activation phase; A-wa-p, amplitude during weight acceptance phase; A-po-p, amplitude during push-off phase; T-ini, time of start of activation; T-max, time of maximum activation; T-total, total time of activation; B, barefoot; S, shod; iEMG units, integrated EMG units (normalised as a percentage of root mean square amplitude); Norm.units, normalised to mean amplitude during the entire gait cycle. * Variables were reported to have statistically significant differences between groups in original study.

Two studies performed comparisons of lower limb muscle function (amplitude and/or timing) [11,27] (Figures 8 and 9A-D). Azevedo et al. [27] reported no significant effects for the amplitude of lateral gastrocnemius at pre-and post-heel strike between cases and controls. Baur et al. [11] showed that the amplitude of lateral gastrocnemius to be significantly reduced during weight acceptance (shod and barefoot) (d = -1.50 and-2.46 respectively) but significantly increased during push-off (shod and barefoot) (d = 0.69 and 1.26 respectively) in cases. Further, the total time of activation of lateral gastrocnemius (shod and barefoot) (d = 0.80 and 1.21 respectively) [11] was significantly increased in cases. Baur et al. [11] investigated medial gastrocnemius function and showed that cases displayed significantly increased amplitude during push-off (shod) (d = 0.86). There were no other significant effects for the amplitude or timing of onset of this muscle (Figure 8).

Azevedo et al. [27] showed that the amplitude of tibialis anterior was significantly reduced at pre-heel strike (100 ms before heel strike) in cases (d = -1.00). Baur et al. [11] showed the amplitude of tibialis anterior during weight acceptance (shod) (d = 1.06) and push-off (barefoot) (d = 1.93) to be significantly increased in cases. Further, the onset of activation of tibialis anterior (shod and barefoot) (d = 0.65 and 0.67 respectively) [11] was significantly increased (delayed) in cases (Figure 9A).

Baur et al. [11] showed the amplitude of peroneus longus during pre-activation (shod) (d = 0.76) and during push-off (barefoot) (d = 0.83) to be significantly increased in cases. Azevedo et al. [27] reported the amplitude of peroneus longus at post-heel strike (100 ms post-heel strike) to be significantly reduced (d = -0.67) in cases (Figure 9B).

Baur et al. [11] investigated soleus muscle function and showed that those with Achilles tendinopathy displayed significantly reduced amplitude during pre-activation (shod) (d = -1.49) and weight acceptance (barefoot) (d = -1.48) but increased during push-off (shod and barefoot) (d = 0.72 and 1.95 respectively). Further, the total time of activation (shod and barefoot) was significantly increased (d = 0.96 and 0.68 respectively) in cases [11] (Figure 9C).

At the hip and knee joints, the amplitude of rectus femoris and gluteus medius post-heel strike (100 ms post-heel strike) were significantly reduced (d = -1.4 and-1.1 respectively) in cases [27] (Figure 9D).

## Discussion

The aim of the present systematic review was to identify, critique and summarise lower limb biomechanical factors associated with Achilles tendinopathy. This review is timely to enhance the development of effective intervention and prevention strategies for the condition. Nine studies [2,11,19,24,25,27,33-35] evaluating lower limb biomechanics in those with Achilles tendinopathy were identified, with eight [2,11,19,24,25,27,34,35] containing sufficient data to complete effect size calculations.

### Quality

In agreement with other studies [30,36,37] that have used Quality Index [31], high inter-rater reliability for the selected items used in this study was found. Methodological quality was varied, with scores ranging between 4 and 15 out of 17. Several studies did not clearly describe participant characteristics (Item 3) [11,25,33,34] or discuss whether participants invited (Item 11) [11,24,25,27,33-35] or recruited were representative of entire population (Item 12) [11,27,33-35]. This limits the ability of any findings to be applied to a broader population. None of the case-control studies [11,24,25,27,33-35] blinded their outcome assessors (Item 15) making it possible that some of the associated results may have been biased. Several included studies did not clearly describe confounding variables (Item 5) [11,19,25,33-35] or adjust for these in their analyses (Item 25) [11,19,33,34]. Additionally, the validity and reliability of outcome measurements used was not reported by any of the studies (Item 20) [2,11,19,24,25,27,33-35]. One study [11] analysed both limbs of each participant, and pooled data for both limbs within the case group, despite participants in the case group having unilateral symptoms. Two case-control studies [33,34] excluded participants that displayed a rigid foot type in the Achilles tendinopathy but not in the control group. This introduces significant recruitment bias into their studies.

### Lower limb kinematics

Abnormal alignment and function of the lower limb, particularly in the frontal plane at the foot and distal leg, is frequently cited as a risk factor for Achilles tendinopathy [8,10,15,23]. Three studies [25,34,35] evaluating frontal plane kinematics of the rearfoot and/or distal leg were identified in this review. The majority of these comparisons were not found to be different between groups (see Figure 1). However, separate studies showed greater eversion range of motion of the ankle in those with Achilles tendinopathy in both shod [34] and barefoot [35] conditions. Further, one study [34] showed reduced maximum lower leg abduction (barefoot) in those with Achilles tendinopathy. These findings suggest that Achilles tendinopathy may be associated with greater movement excursion of the rearfoot during gait and support the original proposition by Clement et al. [10] who hypothesised that greater movement excursion of the rearfoot may create increased tensile stress and subsequent degeneration along the medial aspect of the Achilles tendon [10]. However, these differences need to be considered in light of this review's results showing no significant effects for the majority of frontal plane rearfoot kinematic variables which includes maximum eversion/pronation. Contrary to the tensile stress theory, no evidence was found to support that torsional stress or 'wringing' of the Achilles tendon was associated with Achilles tendinopathy. Two studies [24,35] investigating transverse plane kinematics of the tibia at the ankle and/or knee joints in those with and without Achilles tendinopathy showed no differences between groups. Prospective rearfoot and lower leg motion evaluation is now needed to further understand its possible link to Achilles tendinopathy development.

Three studies [27,34,35] investigated sagittal plane kinematics of the hip, knee and/or ankle joints at a range of instants during stance phase of the gait cycle. Generally comparisons indicated no differences in these parameters between those with and without Achilles tendinopathy, with the exception of reduced maximum ankle dorsiflexion velocity [35] and knee flexion range between heel strike and midstance [27] in those with Achilles tendinopathy. The link between reduced ankle dorsiflexion velocity and Achilles tendinopathy is unclear but it may indicate a compensation strategy to minimise internal loading of the Achilles tendon in those with Achilles tendinopathy. Reduced knee flexion between heel strike and midstance in those with Achilles tendinopathy has been speculated to be a compensation for weakness of proximal hip muscles (e.g., rectus femoris) during eccentric actions, and the reduced impact absorbing motion has been speculated to cause an increase in load within the Achilles tendon [27]. However, future studies are required to determine if there is a relationship between these kinematic changes and increased internal load within the Achilles tendon.

### Ground reaction forces and joint moments

Three studies [11,25,27] performed a large number of comparisons of ground reaction force variables (direction, magnitude and timing) between those with and without Achilles tendinopathy. Overall, there were few differences in the magnitude of the vertical, antero-posterior and medio-lateral components of the ground reaction force variables between those with and without Achilles tendinopathy. However, there were a number of relatively large effects for variables related to the timing of the ground reaction force. Those with Achilles tendinopathy had a greater (delayed) time to the first vertical peak [25], time to minimum peak force [25] and time to maximum medial force [25] but reduced (earlier) time to maximum braking force [25] and time to maximum lateral force [25]. However, the analysed study [25] was a case-control design and participants were symptomatic during testing. It is therefore possible that injured participants may have altered their gait to minimise stress within their Achilles tendons. Future studies are required to determine if these timing differences can cause changes in Achilles tendon loading.

Only one study evaluating joint moments in those with Achilles tendinopathy was identified [24]. Peak external tibial rotation moment was significantly reduced in those with Achilles tendinopathy, suggesting those with Achilles tendinopathy may have reduced torsional stresses within the Achilles tendon. Interestingly, this is contrary to traditional theory [10]. However, it is possible that reduced tibial external rotation moments may be a compensation to reduce stress within the Achilles tendon. Prospective evaluation is now needed in order to adequately understand the association of external tibial rotation moments with Achilles tendinopathy.

### Plantar pressure parameters

Three studies [2,11,19] evaluated the association of a large number of dynamic plantar loading variables with Achilles tendinopathy. Findings showed those with Achilles tendinopathy demonstrated a significantly more laterally directed force distribution beneath the forefoot at forefoot flat (reduced time to peak force at medial heel and medio-lateral force distribution underneath the metatarsal heads at forefoot flat) [2], a significantly more medially directed force distribution during midstance (reduced lateral deviation of the centre of pressure in the rear-and mid-foot) [2,11] and a significantly reduced total forward progression of the centre of force beneath the foot (reduced displacement of the posterior-anterior component of the centre of force at last foot contact, reduced posterior-anterior displacement of the centre of force during forefoot push-off phase, and reduced total posterior-anterior displacement of the centre of force) [2]. Van Ginckel et al. [2] hypothesised that these findings may explain the development of Achilles tendinopathy as follows. First, the lateral foot roll-over pattern during the contact period of gait in those with Achilles tendinopathy may create diminished shock absorption and exert more stress on the lateral side of the Achilles tendon. Second, the more medially directed force distribution during the midstance phase may represent increased midfoot pronation, unlocking the midtarsal joint. This would increase forefoot mobility and impede the ability of the foot to act as a rigid lever during propulsion. Therefore, higher active tensile forces may be transferred through the Achilles tendon during propulsion, leading to tendon strains. This explanation is reflected in findings showing decreased forward transfer of the centre of force in those who developed Achilles tendinopathy [2].

### Lower limb muscle function

Two studies [11,27] compared EMG amplitude and onset timing of a number of lower limb muscles in those with and without Achilles tendinopathy. One study [11] reported a number of differences in the onset timing of lower limb muscles between those with and without Achilles tendinopathy. Notably, the onset of tibialis anterior activity was significantly delayed, and the duration of soleus and lateral gastrocnemius activity was increased in those with Achilles tendinopathy. It is possible that this timing imbalance, particularly the increased duration of activity of the ankle plantarflexors may create prolonged loading of the Achilles tendon and contribute to tendinopathy development. Alternatively, reduced function of tibialis anterior has been theorised to reduce stiffness of the tendon-muscular system in the lower limb and impede its ability to tolerate and absorb impact forces [27]. This could create increased Achilles tendon loading and lead to tendinopathy.

In regards to the amplitude of function of proximal lower limb muscles, one study [27] showed significant reductions in the amplitude of gluteus medius and rectus femoris but not biceps femoris shortly (100 ms) before or after heel strike in those with Achilles tendinopathy. As eccentric contraction of gluteus medius and rectus femoris is important to dissipate forces at the hip and knee respectively during early stance, reduced activity of these muscles may place greater stress at the foot and ankle causing increased Achilles tendon loading. However, conflicting results were reported for the amplitude of the muscles of the distal lower limb, tibialis anterior, peroneus longus and lateral gastrocnemius [11,27]. The inconsistencies in findings may have resulted from differences in study design between the two studies (participants, gait analysed, electrode placement, parameters assessed and processing of data) as well as questionable reliability of lower limb EMG assessment [38,39]. Based on the often conflicting results of these two studies, it is difficult to make inferences concerning the function of lower limb muscles in those with Achilles tendinopathy. Future well-designed prospective studies using reliable and valid assessments of lower limb muscle function are needed.

### Limitations

In addition to the limitations caused by the quality of the included studies described previously, there are several other limitations of this review. All of the included studies analysed running gait only. Given that one third of participants with Achilles tendinopathy are not physically active [6], the findings of this review may not be applicable to these people. There was a predominance of males across included studies, meaning findings from this review may have limited applicability to females. There are a number of biomechanical factors which were not included in this review, either because they have not been previously evaluated (e.g. joint moments at the foot and ankle) or data did not allow effect size calculations. Interestingly, results from the study of Donoghue et al. [33] which was excluded from data analysis (effect size calculations) in this review showed that individuals with Achilles tendinopathy displayed significantly less variation in lower limb kinematics than healthy controls. Only two studies [2,19] included in this systematic review contained a prospective research design, with both investigating plantar pressures. Therefore, with the exception of a number of plantar pressure variables, the ability to distinguish between cause and effect in this review is limited. Sample sizes of included studies were generally small, meaning 95% CIs for effect size calculation were frequently large. This may have erroneously lead to non-significant effect size calculations, even if a true difference between groups existed. Future well-designed and adequately powered prospective studies are required to overcome these limitations.

## Conclusions

Taken together, the findings from this systematic review suggest that those with Achilles tendinopathy have increased eversion range of motion of the rearfoot, reduced maximum lower leg abduction, reduced ankle joint dorsiflexion velocity and reduced knee flexion during gait. Those with Achilles tendinopathy also displayed altered plantar pressures and ground reaction forces and showed a reduced peak tibial external rotation moment. Further, those with Achilles tendinopathy displayed differences in the timing and amplitude of a number of lower limb muscles. Notably, the onset of tibialis anterior activity was significantly delayed, and the duration of soleus and lateral gastrocnemius activity was increased in those with Achilles tendinopathy. In addition, those with Achilles tendinopathy displayed reductions in the amplitude of gluteus medius and rectus femoris shortly before or after heel strike. The findings in regards to plantar loading of the foot and rearfoot eversion range of motion suggest that there are differences in foot function between those with and without Achilles tendinopathy. However, the findings of this review need to be interpreted with caution due to the limited quality of a number of the included studies. Future well-designed prospective studies are required to confirm these findings.

Although the findings of this review need to be interpreted with caution, they may have implications regarding the management of Achilles tendinopathy. They suggest that normalising specific rearfoot kinematic variables, ground reaction force and plantar pressure variables, transverse plane tibial moments and function of specific lower limb muscles may reduce the risk of an individual developing Achilles tendinopathy and/or improve the effectiveness of interventions to manage this disorder. Future well-designed studies are required to determine if interventions such as foot orthoses and/or physical therapy targeting identified differences in those with Achilles tendinopathy are effective at preventing and/or treating the condition.

## Competing interests

SEM is a Deputy Editor of *Journal of Foot and Ankle Research*. It is journal policy that editors are removed from the peer review and editorial decision making processes for papers they have co-authored.

## Authors' contributions

SEM conceived the idea and obtained funding for the study. SEM and CJB equally designed the study, acquired the data, performed the analysis and interpretation of the data and drafted the manuscript. All authors have read and approved the final manuscript.

## Supplementary Material

Additional file 1 **Search strategy and results from each included database**. Search strategy and results from each included database (MEDLINE, EMBASE, Current Contents, CINAHL and SPORTDiscus). Click here for file

Additional file 2 **Checklist for study inclusion and exclusion**. Checklist for inclusion and exclusion of studies. Click here for file
