# Supplementary material for: Lower limb biomechanics during running in individuals with achilles tendinopathy: a systematic review
Source: J Foot Ankle Res. 2011 May 30;4:15. doi: 10.1186/1757-1146-4-15 (PMC3127828; doi:10.1186/1757-1146-4-15)
Supplement: Additional file 1 — Search strategy and results from each included database. Search strategy and results from each included database (MEDLINE, EMBASE, Current Contents, CINAHL and SPORTDiscus). [file 1757-1146-4-15-S1.DOC]

**Additional File 1 - Search strategy and results from each included database**

|  | Search term | MEDLINE | EMBASE | Current Contents | CINAHL | SPORTDiscus |
| --- | --- | --- | --- | --- | --- | --- |
| 1 | achilles | 7433 | 7070 | 4594 | 1421 | 2636 |
| 2 | achilles tendon | 6173 | 5314 | 2810 | 1128 | 2189 |
| 3 | tendoachilles | 37 | 37 | 17 | 6 | 4 |
| 4 | tendo-achilles | 128 | 120 | 72 | 40 | 23 |
| 5 | triceps surae | 1323 | 1116 | 998 | 173 | 412 |
| 6 | or/1-5 | 8598 | 8056 | 5467 | 1573 | 2921 |
| 7 | pain | 365136 | 470690 | 200762 | 91559 | 29696 |
| 8 | tendinopathy | 3730 | 973 | 1046 | 1250 | 768 |
| 9 | tendinitis | 1575 | 4801 | 1494 | 360 | 2264 |
| 10 | tenosynovitis | 2842 | 2605 | 1126 | 275 | 196 |
| 11 | tendinosis | 433 | 544 | 656 | 174 | 269 |
| 12 | tenopathy | 4 | 7 | 3 | 1 | 2 |
| 13 | partial rupture | 314 | 292 | 219 | 43 | 127 |
| 14 | paratenonitis | 38 | 26 | 44 | 4 | 12 |
| 15 | tendovaginitis | 162 | 85 | 32 | 2 | 4 |
| 16 | peritendonitis | 119 | 77 | 42 | 8 | 29 |
| 17 | achillodynia | 56 | 60 | 47 | 5 | 24 |
| 18 | injur$ | 591321 | 638703 | 314647 | 101609 | 98044 |
| 19 | Or/7-18 | 931151 | 1066084 | 497320 | 183093 | 119658 |
| 20 | biomechanic$ | 76288 | 59282 | 26576 | 9966 | 38016 |
| 21 | kinematic$ | 12631 | 15456 | 54861 | 4074 | 7977 |
| 22 | kinetic$ | 537233 | 303119 | 420076 | 3875 | 18558 |
| 23 | motion | 116812 | 102383 | 219581 | 18728 | 24112 |
| 24 | gait | 25466 | 27995 | 16516 | 6501 | 6998 |
| 25 | walk$ | 55378 | 60250 | 65911 | 15580 | 22030 |
| 26 | locomotion | 26637 | 34855 | 18112 | 864 | 4530 |
| 27 | run$ | 97874 | 102360 | 187460 | 12959 | 93028 |
| 28 | or/20-27 | 882094 | 645875 | 940285 | 57720 | 180292 |
| 29 | 6 and 19 and 28 | 783 | 844 | 483 | 292 | 625 |
| 30 | limit 29 to English language | 658 | 713 | 466 | 292 | 572 |
