# Supplementary material for: Lower limb biomechanics during running in individuals with achilles tendinopathy: a systematic review
Source: J Foot Ankle Res. 2011 May 30;4:15. doi: 10.1186/1757-1146-4-15 (PMC3127828; doi:10.1186/1757-1146-4-15)
Supplement: Additional file 2 — Checklist for study inclusion and exclusion. Checklist for inclusion and exclusion of studies. [file 1757-1146-4-15-S2.DOC]

**Additional File 2.** Checklist for study inclusion and exclusion

| **Study inclusion:** |
| --- |
| 1. Prospective cohort or case-control study design  2. Participants described as suffering from mid-portion (2-6 cm proximal to the tendon insertion) *tendinopathy of the Achilles, Achilles tendinitis, tenosynovitis or tendinosis,* *tenopathy, tendinosis, partial rupture, paratenonitis, tendovaginitis, peritendinitis or achillodynia.* |
| 3. Study does not include participants with concomitant injury or pain arising from structures other than the mid-portion of the Achilles tendon. |
| 4. Study evaluates lower limb biomechanics (lower limb kinematics, dynamic plantar pressures, kinetics (ground reaction forces and joint moments) and muscle activity during walking or running. |
| 5. English. |
| 6. Humans as participants. |
| **Study exclusion:** |
| 1. Publication is a review, case-series study, non-peer-reviewed publication, intervention study, letter, opinion article or abstract. |
